# SAMA: A Method for 3D Morphological Analysis

**DOI:** 10.1371/journal.pone.0153022

**Published:** 2016-04-01

**Authors:** Tessie Paulose, Maël Montévil, Lucia Speroni, Florent Cerruti, Carlos Sonnenschein, Ana M. Soto

**Affiliations:** Department of Integrative Physiology and Pathobiology, Tufts University School of Medicine, Boston, Massachusetts, United States of America; University of Tennessee Health Science Center, UNITED STATES

## Abstract

Three-dimensional (3D) culture models are critical tools for understanding tissue morphogenesis. A key requirement for their analysis is the ability to reconstruct the tissue into computational models that allow quantitative evaluation of the formed structures. Here, we present Software for Automated Morphological Analysis (SAMA), a method by which epithelial structures grown in 3D cultures can be imaged, reconstructed and analyzed with minimum human intervention. SAMA allows quantitative analysis of key features of epithelial morphogenesis such as ductal elongation, branching and lumen formation that distinguish different hormonal treatments. SAMA is a user-friendly set of customized macros operated via FIJI (http://fiji.sc/Fiji), an open-source image analysis platform in combination with a set of functions in R (http://www.r-project.org/), an open-source program for statistical analysis. SAMA enables a rapid, exhaustive and quantitative 3D analysis of the shape of a population of structures in a 3D image. SAMA is cross-platform, licensed under the GPLv3 and available at http://montevil.theobio.org/content/sama.

## Introduction

The past couple of decades have witnessed great progress in optical imaging, computational analysis and modeling of biological systems. In the quest for understanding development in higher organisms, biologists are seeking imaging modalities that are increasingly multidimensional by relying on cutting-edge computational approaches to monitor biological phenomena with exceptional resolution, specificity and dimensionality [1–4]. Computerized analyses of images can now overcome the limitations and biases of human assessments with the aid of three-dimensional (3D) images while using *in vitro* 3D tissue cultures.

In the past five years, 3D tissue culture models have greatly advanced our understanding of tissue morphogenesis and pathogenesis of several diseases, including cancer [5–7] by bridging the gap between complex whole animal models and simple 2D cell cultures that lack the architecture, geometry and functionality of the live glandular tissue. A significant contribution of 3D cultures is towards understanding ductal and branching morphogenesis of epithelial cells with respect to its surrounding extracellular matrix (ECM). 3D cultures of tissues including the mammary gland, lungs, pancreas and blood vessels serve as important tools to study the development of organs and tissues by cell-ECM interactions. Several laboratories, including ours, use different types of 3D culture models to understand normal or neoplastic tissue development [6;8–14].

We analyzed images from the 3D gels using presently available commercial and open-source software and found them lacking in options that acquire biologically relevant morphometric parameters in 3D breast tissue. More specifically, these software lacked techniques to accurately analyze elongation, sphericity, lumen formation, and branching, characteristics that make epithelial structures of the 3D model physiologically and morphologically relevant. In this article, we describe a software tool that can provide a rapid and biologically relevant analysis of the 3D culture models of the breast. Here we introduce our open-source SAMA (Software for Automated Morphological Analysis) that takes into account various parameters that define a normal breast while using 3D simulations of breast morphogenesis.

For the sake of simplicity, we have described in this paper the analysis of only one 3D model, specifically a hormone-sensitive 3D culture model of the human breast. In the normal breast, during early development as well as during puberty and pregnancy, the gland responds to hormones, including estradiol, progesterone and prolactin. These hormones regulate mammary morphogenesis by increasing ductal elongation, lateral branching of the ducts, budding, i.e., formation of acini [15] and also lumen formation [16;17]. Given that there is no established breast cell line that responds to mammotropic hormones, research on hormone action in cell culture is mainly done in 2D using hormone-sensitive cell lines derived from pleural effusions obtained from breast cancer patients. Among these cell lines we have chosen the T47D cell line to develop a hormone-sensitive 3D culture of the breast. This model represents the only available model for the study of hormone regulation of epithelial morphogenesis. In this model, the breast cancer T47D cells were embedded in Type I Collagen and these gels were treated with hormones that are physiologically involved in breast morphogenesis. It was observed that the model responds to hormones in ways similar to what is seen in the breast: estradiol (E2) caused elongation, the progestogen, Promegestone (P) caused branching and Prolactin (Prl) caused budding in these structures [11]. The variety of epithelial structures makes this model a suitable choice for testing the power of SAMA in measuring several morphological parameters.

While we have used SAMA to specifically analyze in vitro 3D models of the breast, we believe that developmental biologists in general as well as scientists keen on understanding tissue morphogenesis can use this technique effectively. SAMA is particularly useful for multi-dimensional and multi-parametric analyses and is able to measure the size, shape, lumen formation and quantitative branching of a variety of structures in a high throughput manner.

In the following sections, we will describe the technical features of SAMA, including a description of how images are processed to obtain the final output. SAMA meets the following demands of 3D image analysis: 1) it uses novel algorithms for image analysis that are able to identify, count and analyze crowded 3D structures, 2) it has a versatile design allowing analysis of epithelial phenotypes in several types of matrices, 3) it is open-source and written mostly in R and ImageJ macro language which are simple languages, so the underlying methodology is known and can be modified or improved by users with relative ease, 4) it is fully operated from ImageJ through a user-friendly interface and finally, 5) it is an automated, high-throughput analysis that eliminates the monotony that is typically involved in image analyses (for instance, image formatting, looping several image analysis steps and repeating the analysis without human bias).

## Materials and Methods

### Description of SAMA

SAMA is a novel method by which epithelial structures grown in 3D cultures can be imaged, reconstructed and analyzed with minimum human intervention and bias. It is a powerful and flexible tool for high-throughput morphological and statistical analysis of 3D images. Additionally, we have included an analysis to evaluate the reproducibility of results within and among experimental groups, a critical necessity in biological experiments.

SAMA is a user-friendly set of customized macros with an interactive and user-friendly interface that uses many tools of FIJI (http://fiji.sc/Fiji) [18], an open-source image analysis platform, in combination with a set of functions in R (http://www.r-project.org/), an open-source software tool for statistical analysis. The website for downloading SAMA is http://montevil.theobio.org/en/content/sama. Herein, it is accessible as a FIJI plugin, compatible with Windows, Mac, and Unix along with downloadable source code. In addition, the website includes detailed technical description of the software with sample images for the user to download and learn how to navigate SAMA. Several plugins used in SAMA are available when FIJI is downloaded, while others have to be installed by the user, the details of which are available on the website.

### Image processing

A salient requirement in image analysis is to obtain a homogenized image stack in terms of image resolution and luminosity. 3D structures with homogeneous pixel values that are significantly different from the background are desirable so that a threshold can be applied for segmentation. However, this is difficult to achieve because acquiring images at low magnifications (20X) often results in pixelated and poorly resolved images, leading to artifacts in image analysis. One of the problems we encountered was conjoined structures in crowded areas of the gel due to limited resolution of voxels. Another hindrance was differences in luminosity within the z-stack images (typically the slice closest to the laser will be the brightest and subsequent slices will have decreased intensity, and existing solutions did not work for this type of data set as they rely on more homogenous media [19]. This resulted in dim structures in slices that were farther away from the laser. To solve issues of imaging irregularities, we included several steps of 3D filtering that take into account the 3D information of the signal and reduce the noise and artifacts in images. Briefly, we first remove the local background of the images and then, the images are subjected to several filters including 3D Median, Variance, Minimum and Maximum filters alone or in combination. These filters ensure that most of the noise is removed, while the shape of the structure is maintained. Additionally, a 3D Gaussian blur filter is used to smooth the surface of the structures by normalizing the intensity of pixels with reference to neighboring pixels. We compensate the loss of luminosity for deeper slices of the stack on the basis of the running average of the maximum luminosity of each slice, so that for each slice, the previous, current and next slice are taken into account to assess and compensate for the loss of luminosity. In addition, for the morphological analysis, using 2D fill holes, we fill the structures to avoid interference from the lumen. Using these filters in image processing is important for two reasons; first, homogenized pixels in the structures have similar threshold intensity, thus allowing an accurate morphological analysis, and second, lumen in the homogenized structures can be accurately segmented and analyzed. An optional step for images with crowded structures is to separate out structures that were merged because of poorly resolved voxels. To achieve this we subjected the image to 3D Gradient filter process, which demarcates the edges of the structures. This image with demarcated structures is then subtracted from the original image, thus getting rid of unwanted pixels between structures. This step ensures an accurate count and morphological measurement of individual structures. However, in images where crowded structures are not an issue, these steps can be left out of the algorithm. Image processing steps described here are incorporated at several stages in the SAMA-images section of the code depending on the end-point needed.

A drawback of most commercial software is the inability to adapt new parameters that result from either including additional steps for image processing or calculating new features of epithelial structures. In contrast, SAMA's modular algorithm and open-source code allows users the choice of including additional image processing steps or novel phenotype measurements as needed. As an example of giving users the option to choose steps during image processing, we used images in which most of the background noise was removed by using wavelet techniques [20], a de-noising plugin. However, de-noising was excluded from the main SAMA framework and was run separately because it required more memory to function than what a typical personal computer has, the absence of which may cause the program to fail. With SAMA, users have the freedom to run de-noising or an alternative algorithm based on bilateral filtering as a stand-alone batch process function depending on the user’s computer memory (the stand-alone de-noising batch process is included).

### User-interface

**Fig 1:**
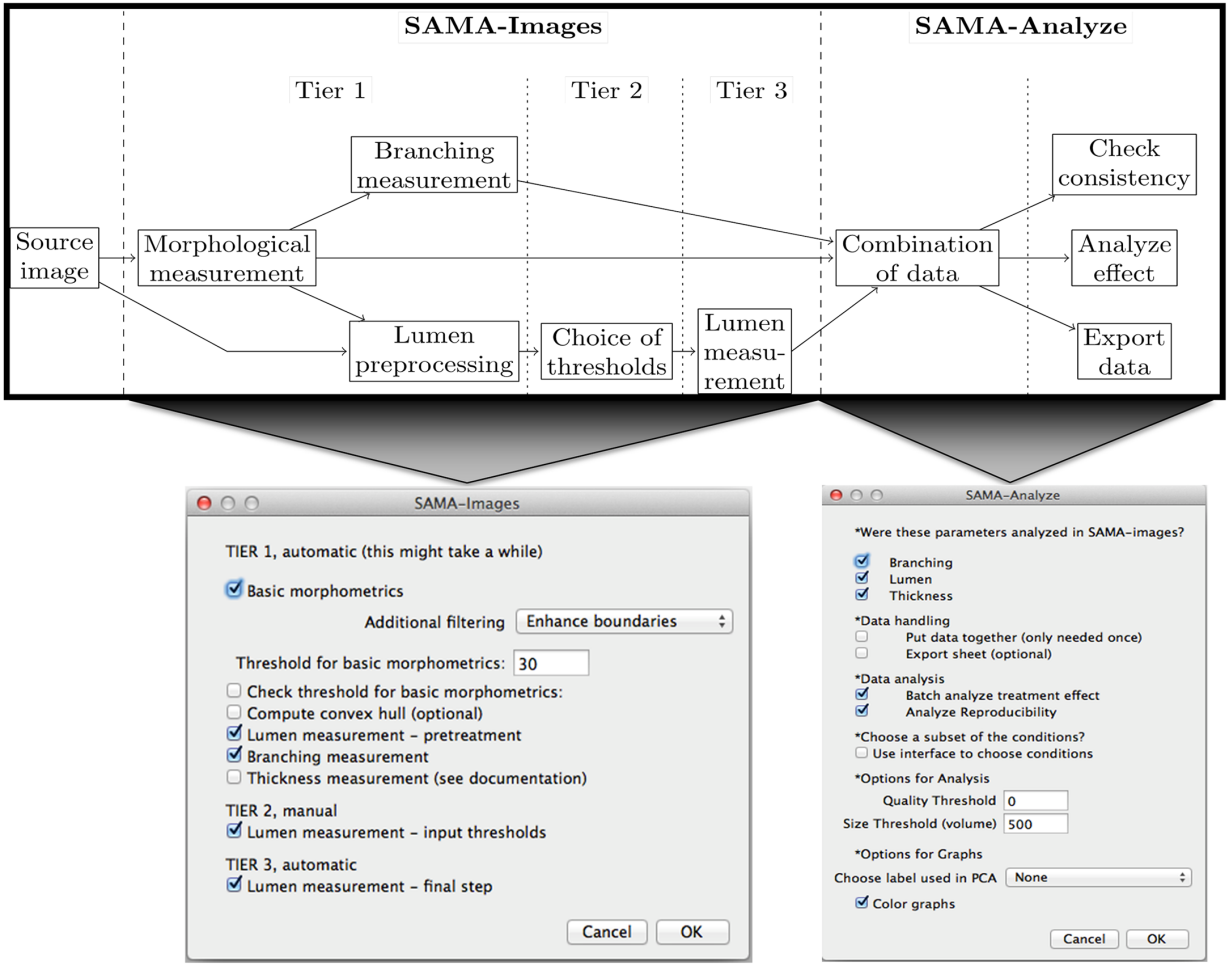
Flow-chart and user-interfaces of SAMA. General algorithm of functions in SAMA (top panel). The two main interfaces of SAMA—SAMA-Images (bottom left) and SAMA-Analyze (bottom right).

**Fig 2:**
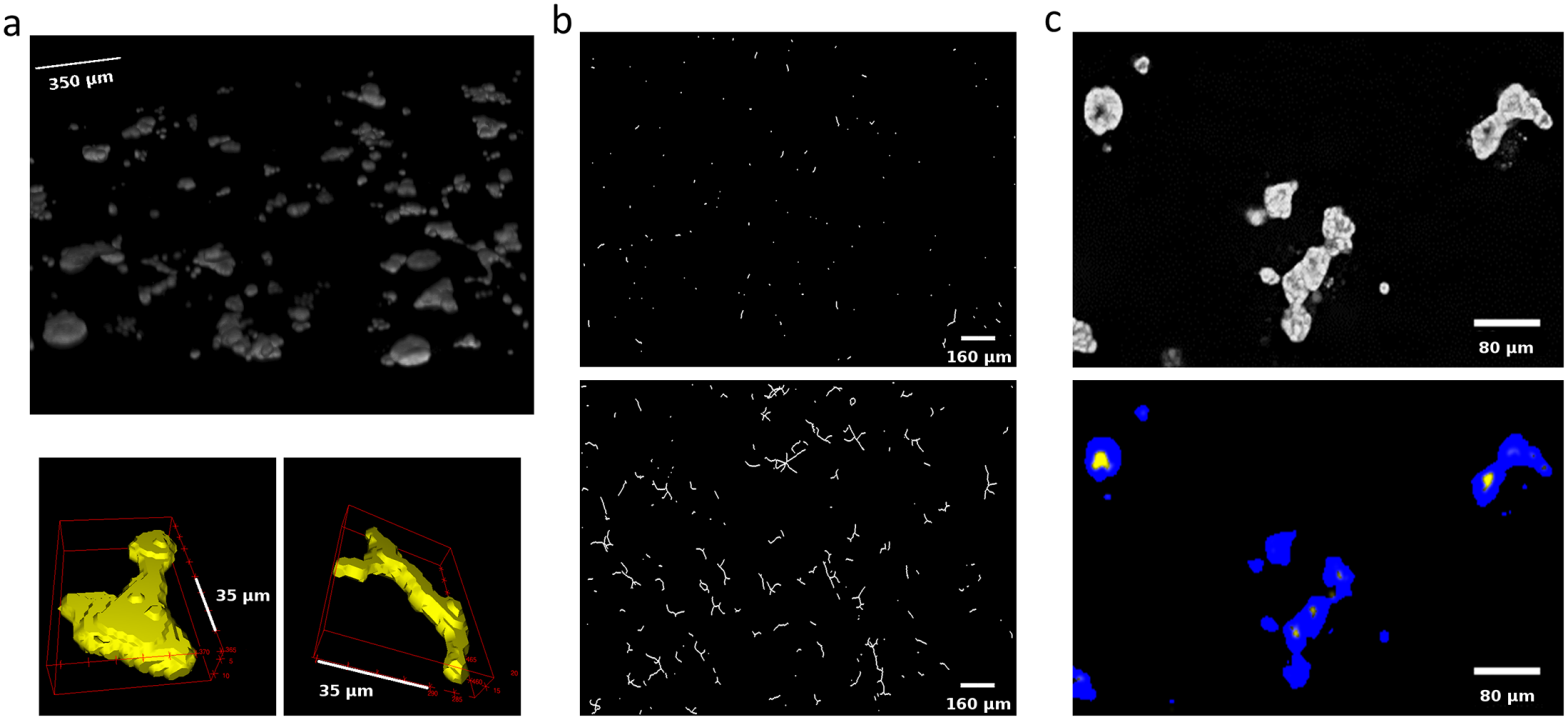
Basic morphometrics, branching and lumen analysis in the 3D hormone-sensitive breast model. (a) An overview of all 3D structures (top) and individual 3D structure showing budding from E2+Prl (bottom left) and branching from E2+P (bottom right) treatment groups. (b) Branching of structures in E2 alone (top) and E2+Prl (bottom). (c) Raw image of structures with lumena (top) and the processed image of structures with segmented lumena (bottom).

**Fig 3:**
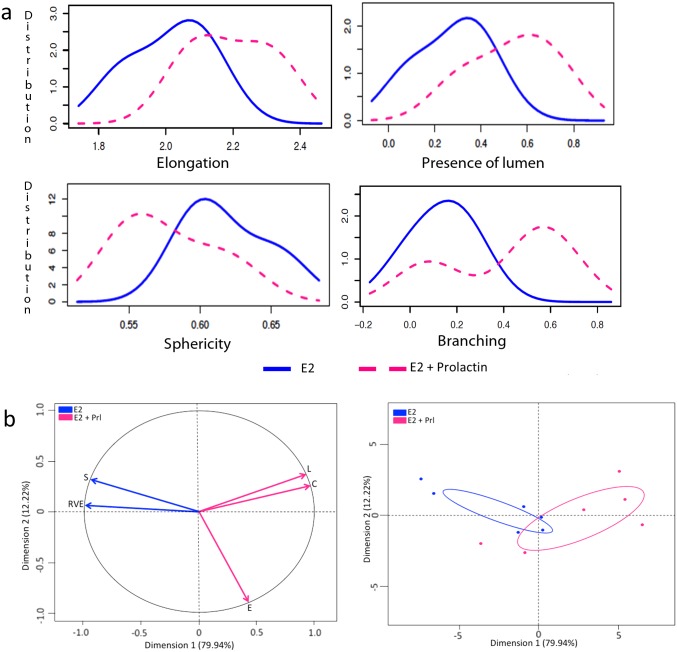
Morphometric analysis of structures in E2+Prl treatment group. (a) Graphs represent 3D parameters that were calculated using the formula shown in Table 1; elongation (top left), presence of lumen, (top right), sphericity (bottom left) and branching (bottom right). X-axis represents the mean of the parameters and Y-axis represents the distribution of the structures measured in three experimental replicates. Blue (solid) line represents E2 alone treatment and pink (dashed) line represents E2+Prl. (b) Principal component analysis (PCA) shows variance in the data by analyzing correlated variables between E2 and E2+Prl. Factor map with vectors represent highly correlated variables as Dimension 1 and 2 on the x and y axes respectively. Correlated variables sphericity (S) and ratio volume ellipsoid (RVE) in E2 treatment shown with blue arrows while correlated variables elongation (E), number of lumen (L) and complexity (C) in E2+Prl shown with pink arrows (left). Graph shows distribution of the structures in each treatment group, E2 (blue) and E2+Prl (pink) with correlated variables (right).

**Fig 4:**
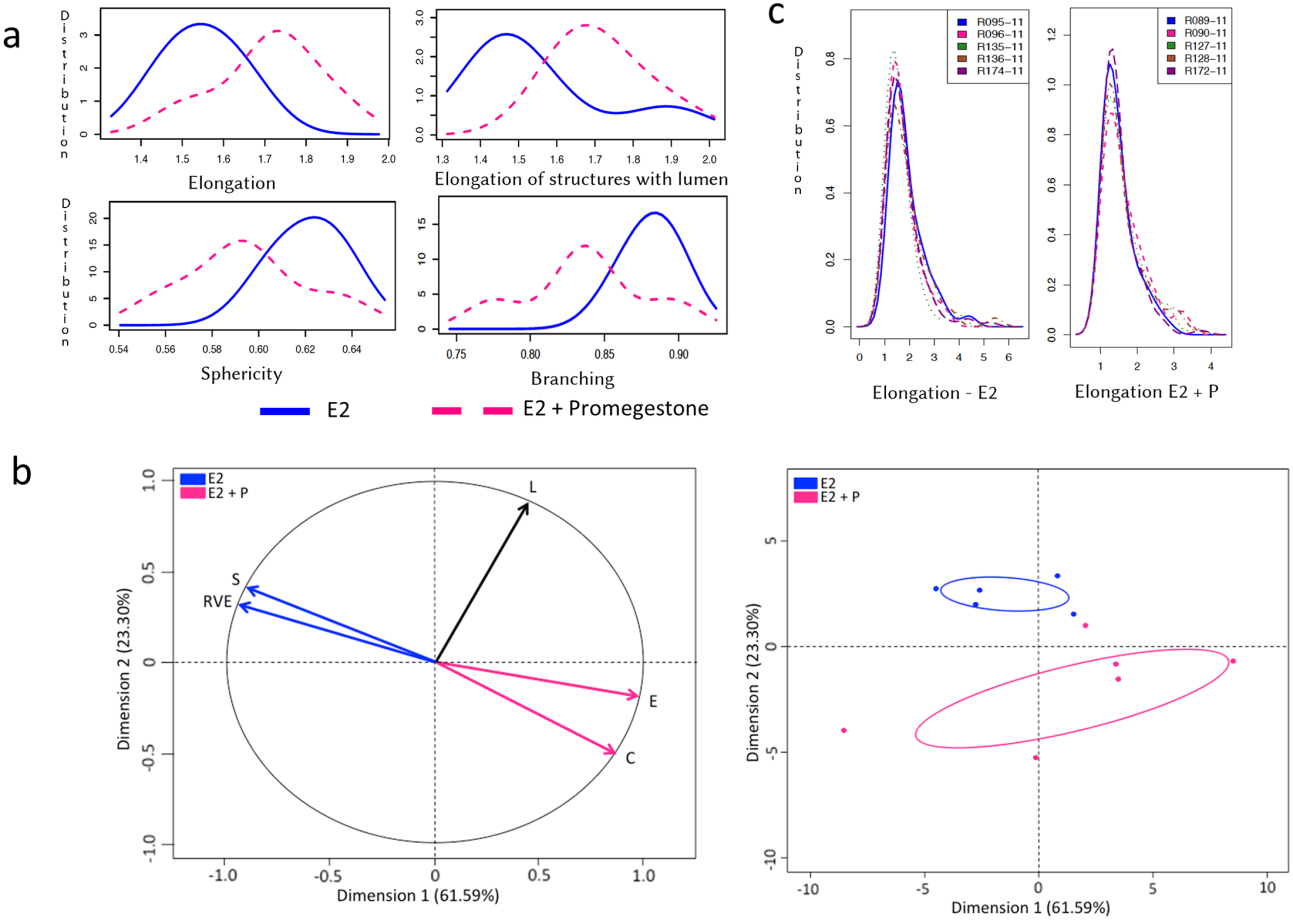
Morphometric analysis of structures in E2+P treatment group. (a) Graphs represent 3D parameters that were calculated using the formula shown in Table 1; elongation (top left), elongation in structures with lumen (top right), sphericity (bottom left) and branching in terms of ratio volume ellipsoid (bottom right). X-axis represents the mean of the parameters and Y-axis represents the distribution of the structures measured in three experimental replicates. Blue (solid) line represents E2 alone treatment and pink (dashed) line represents E2+P. (b) Principal component analysis (PCA) shows variance in the data by analyzing correlated variables, sphericity (S), ratio volume ellipsoid (RVE), elongation (E), complexity (C) and number of lumen (L) between E2 and E2+P. Factor map with vectors represent highly correlated variables as Dimension 1 and 2 on the x and y axes respectively. Vector colors, blue, pink and black correspond to the trend in the distribution of structures in E2, E2+P and both respectively (left). Graph shows distribution of the structures in each treatment group, E2 (blue) and E2+P (pink) with correlated variables (right). (c) Graphs represent reproducibility in the measurement of elongation in structures of E2 (left) and E2+P (right). X-axis represents the mean of the parameters and y-axis represents the distribution of the structures measured in five experimental replicates shown as a number code.

The SAMA plugin has two main parts—“SAMA-image” and “SAMA-analyze” (Fig 1). SAMA-image allows users to analyze 3D structures in three tiers. Tier 1 is fully automated and is a mandatory step for subsequent tiers. Users have a choice to carry out basic morphometrics, complexity (branching analysis) and lumen analysis either simultaneously or individually. Basic morphometric analysis includes counting all the structures in the 3D image and saving the information in a text file. The data obtained from this analysis contain several morphologically relevant parameters (explained in the following section) that define the shape and size of the structure (Fig 2A). The image files are batch-processed from a user-specified source directory wherein output files are stored as well. The plugins used in this tier for analyzing basic morphometrics are 3D Object counter and 3D ROI manager [21;22]. In complexity analysis, branches of the structures are quantified and these data are stored as separate files in the source directory. Briefly, the structures are skeletonized (structures stripped to the bare minimum pixels while preserving their overall shape) [23]and SAMA identifies each skeleton with the corresponding structure (Fig 2B). This enables the user to quantify the complexity of the skeleton (number of branches and cumulated length of branches). Lumen analysis spans Tiers 1, 2 and 3. In Tier 1, the images are pre-processed for lumen analysis using several filters and the resulting images are then used in Tier 2. In Tier 2, the user specifies the threshold for processed images that segments lumen in the structures, a value that varies from image to image and hence, cannot be automated. In Tier 3, lumena are identified by the difference in pixel intensity between the surrounding cells and the hollow space in the epithelial structure. Multiple lumena are detected as regions, which appear darker than their surroundings, forming a hole, which are then segmented (Fig 2C). The segmented lumena are counted and analyzed by 3D object counter and 3D ROI manager plugins, the corresponding structures are also identified. Every lumen thus obtains an identity of its own with 3D parameters. All parameters obtained in Tiers 1, 2 and 3 are then statistically analyzed in SAMA-analyze. SAMA-analyze operates the part of SAMA written in R through the interface in ImageJ, which contains two main parts—Data handling and Data analysis. Data handling consolidates all the data gathered from Tiers 1, 2 and 3 of SAMA-images into a single spreadsheet for analysis. Here, the user has the option to include or exclude lumen and branching analysis from basic morphometric analysis. SAMA-analyze generates an exhaustive report, which compares shape and morphology of epithelial structures obtained in the different conditions/treatments. Biologically relevant parameters (Table 1) (S1 Fig) of each experimental replicate are represented as the mean, standard deviation, coefficient of variation and median with respect to the distribution of structures in each group. This report includes a graph for each quantity and function of the estimated distribution of the replicates and a boxplot of the same data. ANOVA is performed to assess the significance of the experimental conditions and Wilcoxon rank test is also performed in the case where only two conditions are present. An overview of the results is obtained by principal component analysis (PCA) (Figs 3B and 4B), which enables to identify the variables that have a greater influence in the variance of the samples. This technique enables the user to represent a data set with many parameters by defining new dimensions (which are combinations of the original variables) and represents a simple way of understanding the variance of the data set. This is helpful when many parameters are correlated to each other. In the case of SAMA, some data sets, for example, lead to a size related quantity and a shape related quantity. Note however, that the definitions of such quantities are obtained *de novo* for each dataset. Note also, that parameters that are biologically relevant but are not correlated with other parameters are usually not taken into account by this analysis because it focuses on trends present in many quantities.

**Table 1:** List of quantitative parameters and their corresponding mathematical definitions.

Parameter	Abbreviation	Description/formula
Elongation	Elon1	Long axis / Middle axis of the 3D structure
Flatness	Elon2	Middle axis / Short axis of the 3D structure
Sphericity	S	[(36*π*Volume^2^)/Area^3^]^1/3
Relative Lumen Volume	RLV	Volume of the structures with lumen/Volume of the lumen
Lumen	L	Number of structures with lumen/Number of structures
Quality	Q	Elon1 –Elon2 + (5*RLV)
Ratio Volume Ellipsoid	RVE	Volume of structure/Volume of fitted ellipsoid
Complexity	C	Sum of length of all branches

### Computer generated images

In order to validate SAMA, we tested our software on computer generated (CG) stacks (S2 Fig). The stacks were generated with 40 ellipsoids, which mimic an image containing 40 epithelial structures in a z-stack obtained by confocal microscopy of 3D cultures. These CG structures are at random positions, orientation and sizes in the three axes. Lumena are smaller structures constructed with a randomly chosen radius within the larger structures. The reference condition (C1), are ellipsoids that contain a lumen, have similar probability distribution in terms of size and orientation towards any of the axes. Condition C2 was designed to differ from C1 in that the maximum size of the ellipsoid oriented in one axis of the ellipsoid is twice the size that is oriented towards the other axes. Condition C3 differs from C1 because it was made with each structure having only a 50% chance of having a lumen. Condition C4 contains structures with two ellipsoids positioned perpendicularly, to test the ability of SAMA to detect structures with a higher complexity. For each condition, we generated six stacks.

### Three-dimensional cultures

The epithelial cells and components used in the 3D cultures of the breast used for validation of SAMA have been previously described [11]. Briefly, cells were suspended in a final volume of 1.5 mL of collagen (1 mg/ml) and poured into 12-well plates (Becton Dickinson). The mixture was allowed to congeal for 30 min at 37°C and 1.5 mL of the CD-FBS or CD-FBS medium containing estradiol alone (10^−10^,10^−9^M), or in combination with promegestone (10^−10^M), or prolactin (10^−7^M)was added to each well. Gels were detached [10] and cultures were maintained for two weeks for cell organization analysis and the medium was changed every two days. After two weeks, gels were fixed overnight in 10% phosphate-buffered formalin and stained with Carmine-alum.

### Image acquisition

Whole-mounted gels stained with carmine alum were imaged using a Zeiss LSM 510 system (Carl Zeiss MicroImaging, Inc.). Samples were excited with the HeNe 633nm laser as the Carmine dye fluoresces at this wavelength. Images were acquired at 20X magnification using a MultiTime macro, which enables automated imaging of multiple frames of z-stacks, which are then stitched using 20% overlap of images to obtain 5 X 3 image tiles.

### Statistical analysis

The results we discuss are obtained both by ANOVA and Wilcoxon rank test and the p-value reported is the largest between the two values obtained. All statistical analyses are performed with cran R. The PCA analysis is performed with the R package FactoMineR.

### Imaging parameters

A description of the parameters and the formula that were used to give an in-depth morphological analysis of the epithelial structures is described in Table 1. The basic morphometric parameters generated by 3D ROI manager plugin used in SAMA’s program are elongation, flatness, sphericity and volume. For lumen analysis, we incorporated two parameters, the presence of a lumen and the relative lumen volume (ratio of the volume of the lumen to the volume of the structure). Quality is a derivative quantity, which takes into account the elongation of the structure and relative lumen volume. Morphologically relevant epithelial structures in 3D gels are ones that are optimally elongated, not very flat and contain a lumen. These features resemble those of a normal breast epithelium. Hence, a positive value for quality would indicate normal structures while negative values for quality would indicate that the structure is flat. For branching analysis, we use parameters that are generated by 3D ROI manager, namely ratio volume ellipsoid (RVE) as well as a parameter we generated, namely complexity. RVE is the ratio of the volume of the structure to the volume of the ellipsoid fitted around the structure. Hence a value of 1 will indicate a perfect ellipsoid or sphere whereas values close to 0 will indicate a convoluted structure, indicating the presence of branches or protrusions. We assess skeletonized structures with several complexity quantities: whether there is more than one branch, the number of branches, and the cumulated lengths of the branches. The higher the value of complexity, the more convoluted or branched the structure would be.

## Results and Discussion

### Validation by computer generated images

To ensure that SAMA is functional, we validated the software using computer generated model images. The model images were constructed to test several analytical functions of SAMA. SAMA provides the expected outputs from our designed conditions (S2 Fig). The analysis shows that C2 has more elongated structures than C1 (p = 0.002). In C3, there are more structures with lumen (p = 0.016) and overall structures have bigger lumena (p = 0.002). Condition C4 has structures that are more complex than C1 for all of our measures of complexity (p<0.026). As a result, SAMA performs as expected in computer generated images.

### Biological Validation and Analysis

To validate SAMA, we used images from a hormone-sensitive 3D culture of the breast [11]. We chose to analyze gels that have previously been analyzed using 2D image measurements as well as 3D computational modeling and visualization using the commercial software package, Volocity [11].

We observed that T47D cells that were not exposed to hormones did not develop any epithelial structures and hence, these gels were excluded from our analysis. In the 3D gels analyzed here by SAMA, the cells responded to E2 by proliferating and forming elongated and spherical 3D structures. Further, treatment of cells with E2+P and E2+Prl caused significantly higher elongation of structures in the gels compared to E2 treatment alone (Figs 3A and 4A). These results are consistent with previously published results from 2D analysis [11]. In addition to these data, we were able to analyze new features including lumen formation and branching of these structures. We found that in E2+Prl treatment group, structures had higher number of lumen compared to E2 alone (Fig 3A). In E2+P treatment group, structures with lumen had significantly higher elongation than E2 alone (Fig 4A). Similarly, we observed that E2+Prl and E2+P gels had higher branching in structures with hormone combinations than E2 alone (Figs 3A and 4A). The PCA factor map and graphs give a comprehensive analysis of correlated variables, which indicate the diversity of structures formed in E2 treatment alone and E2 + hormone treatment (Figs 3B and 4B). These data, thus give us new quantifiable information regarding lumen and branching of structures in these treatments, features that could only be qualitatively analyzed previously. Additionally, SAMA analyzes the reproducibility of results obtained within and between treatment groups. Specifically, SAMA is able to measure reproducibility and variability in the tissue culture system by calculating the mean of biologically relevant parameters and plotting this as a distribution curve. As an example, we have shown the distribution of one such parameter, namely elongation, as this is the distinguishing feature between ducts and acini. The graph that SAMA generates shows whether the distribution curve of each experimental replicate of a given sampling group superimposes on each other. Superimposition of these curves would indicate that the structures are not variable within treatment replicates, suggesting that the results were reproducible between experiments (Fig 4C). This enables the user to understand variability in 3D morphogenesis that is inevitable in biological experiments and observe how these inconsistencies distribute among the experimental groups and replicates.

### SAMA versus current software

Currently, there are several commercial as well as open-source software packages available that combine image acquisition and analysis (Table 2). Among them, Imaris (Bitplane), Volocity (Perkin-Elmer), Image-Pro (MediaCybernetics), Amira and Metamorph (Molecular Devices) are examples of commercially available software. CellProfiler [24], V3D [25], BioImageXD [26] and NEMO [27] are examples of open-source software platforms for the analysis of biological images. The advantage of commercial software is that it is tailor-made for the imaging system purchased, with the availability of customer support and warranties from the company. Their drawbacks include the high costs of purchasing hardware-software bundles and most critically, the inability to know and modify the proprietary codes. This prevents researchers from knowing the details of the computing processes and modifying them to acquire specific and biologically relevant end-points. While these are aspects where open-source software has an edge over commercial software, the presently available open-source software packages have limitations as well. Existing open-source software enables basic morphometrics (structural parameters only), but there are limited options available for an exhaustive, high throughput, biologically relevant multi-parametric image analysis bundled with statistical analysis that takes into account reproducibility among experiments. For example, there are limited software packages that can carry out in-depth analyses (a combination of morphological, statistical and reproducibility analyses of shape, size, lumen formation and branching) of 3D epithelial structures in models of biological systems. To remedy this latter inconvenience, we developed SAMA to 1) obtain an exhaustive and comprehensive analysis of tissue morphogenesis in 3D culture models of the breast, 2) to gain a deeper understanding of biological variability in tissue morphogenesis in 3D tissue cultures, 3) to acquire a rapid, automated and unbiased analysis of every epithelial structure in the 3D images that span a large sampling area and, 4) to combine imaging analysis with a meticulous analysis of the experimental replicates, thus ensuring reproducibility in the data obtained from the 3D cultures.

**Table 2:** Comparison between SAMA and other software packages that combine image acquisition and analysis.

Software	Open-source	Biological use	High-throughput	3D morphometrics	Lumen analysis	Branching analysis	Statistical analysis	Reproducibility analysis
SAMA	✓	Morphological analysis	✓	✓	✓	✓	✓	✓
Cell Profiler	✓	Cell morphology	✓	X	X	X	X	X
V3D	✓	Mostly neuro-oriented morphological analysis	✓	✓	X	X	X	X
NEMO	✓	Neuron Morphology	✓	✓	X	✓	X	X
BioImage XD	✓	Morphological analysis	✓	✓	X	X	X	X
Imaris	X	Morphological analysis	✓	✓	X	X	X	X
Volocity	X	Morphological analysis	X	✓	X	X	X	X
Image-Pro	X	Morphological analysis	✓	✓	X	X	X	X
Amira	X	Morphological analysis	✓	✓	X	X	X	X

3D tissue cultures are increasingly becoming a source of new information regarding tissue morphogenesis, which can lead to a more detailed understanding of carcinogenesis in tissues such as the breast. However, lack of proper methodology and software prevents researchers from getting an in-depth analysis of a large population of 3D structures in images. Researchers tend to choose a few epithelial structures from a large heterogeneous population of 3D structures to represent the outcome of a given experimental condition [12;28]. Heterogeneity in the distribution and shape of epithelial structures in a 3D gel is a phenomenon that we have observed within each gel as well as over a given period of time [10]. With such a heterogeneous population, it is imperative that a large sampling size be taken into consideration to analyze and represent the global experimental outcome. Hence, SAMA was developed keeping in mind the need to have an exhaustive analysis of the 3D sample so that biologists are not constrained to choosing a restricted number of structures that require time-consuming manual analysis and may not truly represent the overall outcome of their experimental conditions.

## Summary

We have compared SAMA to existing commercial and open-source software. Specifically, we compared biologically relevant end-points that SAMA analyzes to those analyzed by other software (Table 2). These comparisons of biological end-points hold greater bearing for our analyses than comparing imaging features and software intricacies. The open-source SAMA is our contribution to the growing community of scientists working with 3D cultures who want to obtain a comprehensive, multi-parametric and biologically relevant analysis of 3D structures of the tissue. We encourage scientists to not only download and use SAMA, but also innovatively improvise its code to help in this endeavor.

## Supporting Information

S1 Fig Visual representation of parameters. This figure is a representation of how elongation and branching are calculated by SAMA. The measurement of elongation as shown in Panel A has been explained using a 3D cylinder (representing an epithelial structure) in which the three axes have been labeled as L, M and S. Elon1 is the ratio of the long axis to the middle axis (L/M). Elon 2 is the ratio of the middle axis to small axis (M/S). Elon1 > Elon2 would mean that the epithelial structure is elongated. Elon2 > Elon1 would mean the epithelial structure is flat. Elon1 = Elon2 would mean that the epithelial structure is spherical. Branching was measured in two ways, namely Complexity and Ratio Volume Ellipsoid (RVE). Panel B explains how complexity is measured. The shapes “Y” and “I” represent branched and single (no branching) epithelial ducts. Complexity is the measurement of the length of structures. The sum of the three arms (represented by the three black lines inside the pink structures) of “Y” is calculated to give the total length of “Y”. Similarly the length of “I” is calculated. The structure with greater length is more complex than ones with smaller length. Panel C explains how RVE is calculated by drawing an ellipse around “Y” and the letter “O” (representing an acini). RVE is the ratio of the volume of the structure to the volume of an ellipsoid fitted around the structure. Log RVE is close to 0 for spherical structures and close to 1 for branched structures.
(TIFF)

S2 Fig Validation of SAMA. Panel A: Computer generated images of four test conditions. Each one is a single representative image of the complete stack. Panel B: Resulting SAMA output of four parameters each compared to the reference condition (C1).
(PDF)
