# Supplementary figures and images for: SAMA: A Method for 3D Morphological Analysis
Source: PLoS One. 2016 Apr 1;11(4):e0153022. doi: 10.1371/journal.pone.0153022 (PMC4818086; doi:10.1371/journal.pone.0153022)

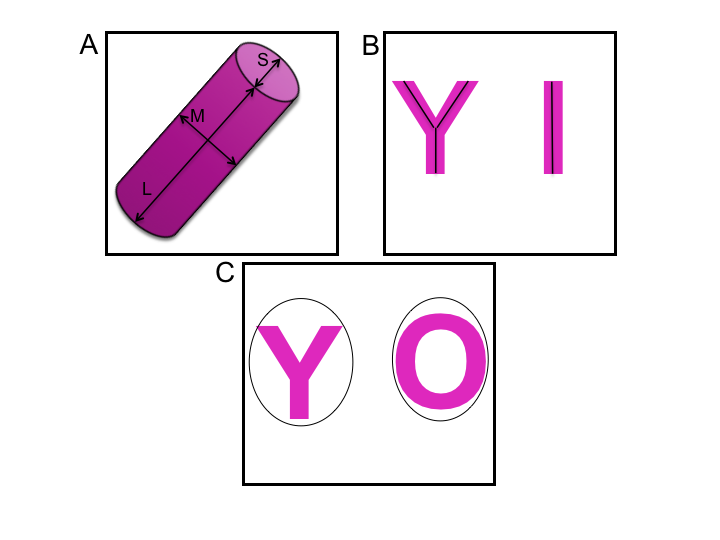

Supplement: S1 Fig — This figure is a representation of how elongation and branching are calculated by SAMA. The measurement of elongation as shown in Panel A has been explained using a 3D cylinder (representing an epithelial structure) in which the three axes have been labeled as L, M and S. Elon1 is the ratio of the long axis to the middle axis (L/M). Elon 2 is the ratio of the middle axis to small axis (M/S). Elon1 > Elon2 would mean that the epithelial structure is elongated. Elon2 > Elon1 would mean the epithelial structure is flat. Elon1 = Elon2 would mean that the epithelial structure is spherical. Branching was measured in two ways, namely Complexity and Ratio Volume Ellipsoid (RVE). Panel B explains how complexity is measured. The shapes “Y” and “I” represent branched and single (no branching) epithelial ducts. Complexity is the measurement of the length of structures. The sum of the three arms (represented by the three black lines inside the pink structures) of “Y” is calculated to give the total length of “Y”. Similarly the length of “I” is calculated. The structure with greater length is more complex than ones with smaller length. Panel C explains how RVE is calculated by drawing an ellipse around “Y” and the letter “O” (representing an acini). RVE is the ratio of the volume of the structure to the volume of an ellipsoid fitted around the structure. Log RVE is close to 0 for spherical structures and close to 1 for branched structures. (TIFF) [file pone.0153022.s001.tiff]

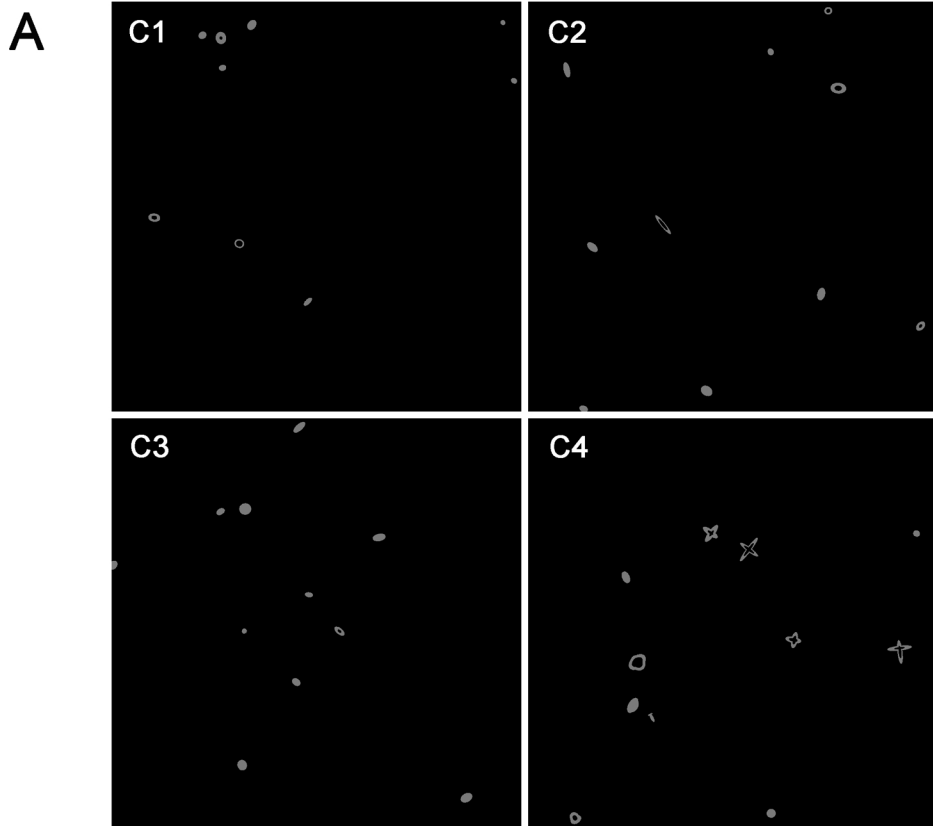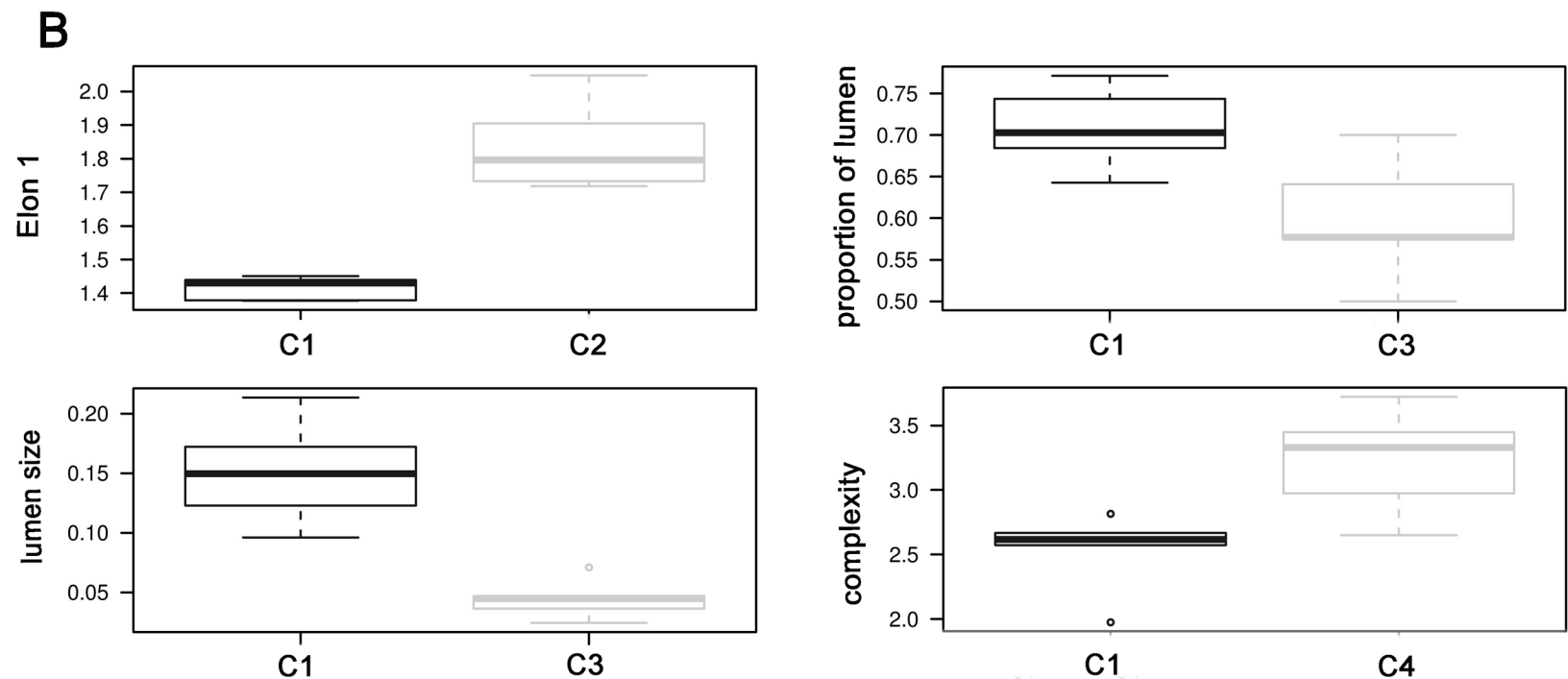

Supplement: S2 Fig — Panel A: Computer generated images of four test conditions. Each one is a single representative image of the complete stack. Panel B: Resulting SAMA output of four parameters each compared to the reference condition (C1). (PDF) [file pone.0153022.s002.pdf]
